# Heterogeneity of Ex Vivo Tumor Responses in Ovarian Cancer Tissues

**DOI:** 10.1002/cnr2.70554

**Published:** 2026-06-14

**Authors:** Chiara Maestri, Ivan Trus, Rajeshwar Nitiyanandan, Jahanvi Kumar, Ricardo J. Parker, Orazio De Tommasi, William Cance, Christian Apfel

**Affiliations:** ^1^ SageMedic Corp Redwood City California USA; ^2^ International Institute of Molecular and Cell Biology Warsaw Poland; ^3^ National University San Diego California USA; ^4^ Department of Women and Children's Health, Clinic of Gynecology and Obstetrics University of Padova Padova Italy; ^5^ University of Arizona College of Medicine Phoenix Arizona USA; ^6^ University of California San Francisco San Francisco California USA

**Keywords:** ex vivo drug testing, functional profiling, ovarian cancer, precision oncology, tumor heterogeneity

## Abstract

**Background:**

Advanced‐stage ovarian cancers exhibit significant variability in responses to standard chemotherapy ranging from platinum‐refractory to platinum‐sensitive cancers. While genomic testing reveals tissue heterogeneity, it rarely provides actionable data for guiding therapy.

**Aims:**

To evaluate the heterogeneity of ex vivo drug responses in primary ovarian cancer tissues using a functional profiling platform, and to assess the platform's potential for guiding personalized therapy selection within current clinical practice.

**Methods and Results:**

A functional profiling platform that creates 3D microtumors from fresh biopsies was used to enable ex vivo assessment of drug efficacy in a physiologically relevant model. Interpatient heterogeneity was quantified as the statistical variability of the EC_50_ values, calculated using the ratio of upper and lower interdecile values. Seventeen ovarian cancer tissues were processed and exposed to NCCN‐recommended drugs and drug combinations. Drug efficacy demonstrated significant interpatient heterogeneity (*p* < 0.001). Carboplatin and paclitaxel alone exhibited moderate heterogeneity (54‐fold and 42‐fold difference), while their combination showed higher heterogeneity (3880‐fold difference), suggesting synergy in certain samples but not in others. Gemcitabine exhibited the highest single‐agent heterogeneity across the cohort (1504‐fold difference), whereas doxorubicin and olaparib demonstrated more consistent responses but limited efficacy in many samples (20‐fold and 16‐fold difference).

**Conclusion:**

These findings align with the clinically observed variability in patient responses, highlighting the platform's potential to optimize therapy selection and support patient‐centric care within the standard of care for ovarian cancer patients.

## 1 Introduction

Ovarian cancer remains a significant clinical challenge, particularly in advanced stages where therapeutic outcomes vary widely among patients. While most ovarian cancer patients initially respond favorably to the standard carboplatin and paclitaxel regimen, now also increasingly utilized in the neo‐adjuvant setting to improve surgical outcomes, a subset of patients derives no benefit [1, 2]. For these patients, the side effects of ineffective chemotherapy, coupled with the delay in surgery, may result in worse outcomes compared to immediate surgical intervention [3]. Similarly, in the adjuvant setting, some ovarian cancers recur within six months and are usually classified as platinum‐resistant [4]. Furthermore, even among patients with cancers deemed platinum‐sensitive, recurrence remains frequent, contributing to a poor overall prognosis and 5‐year survival rate for advanced‐stage ovarian cancers of about 30% [5]. These challenges highlight the need for better tools to characterize the sensitivity and resistance profiles of each tumor and to better personalize the patient's cancer treatment.

The NCI's MATCH trial, led by Flaherty and colleagues in 2020, revealed that only one in four patients can be matched to a targeted therapy based on their genomic driver mutations, emphasizing the limitations of genomic profiling in guiding effective treatments [6]. Additionally, the extensive intra‐tumoral genetic heterogeneity and cellular plasticity, underscore the complexity of tumor evolution, and contribute to the clinical tumor resistance [7, 8].

Although genomic testing has provided insights into ovarian cancer biology, particularly in cases with BRCA mutations or homologous recombination deficiency (HRD), these molecular diagnostics often fall short of capturing the “*functional”* heterogeneity and the tumor microenvironment, which play a critical role in determining treatment efficacy [9]. This gap is especially pronounced in high‐grade serous ovarian cancer [10] which is included in this study.

Functional profiling has the potential to overcome the limitations of genomic testing by directly assessing tumor sensitivity and resistance to therapies in a physiologically relevant ex vivo setting [11, 12, 13]. Early efforts, such as clonogenic assays, showed strong correlations with clinical outcomes but faced significant practical challenges, including unreliable colony formation and prolonged assay times spanning weeks to months. Additionally, many functional assays relied on two‐dimensional monolayer cultures, which failed to adequately capture the complexity of the tumor microenvironment, including cell–cell and cell‐matrix interactions critical to therapeutic responses. These limitations hindered the widespread adoption of functional profiling, despite its promise to provide actionable insights that could better inform personalized cancer treatment strategies. Currently, the NCCN ovarian cancer guidelines state that chemosensitivity and chemoresistance assays lack sufficient evidence to replace standard‐of‐care chemotherapy, although they may be considered in specific clinical scenarios, such as recurrent or refractory disease with limited therapeutic options [14].

To address the limitations of the forementioned approaches, we developed an internally validated functional profiling platform for clinical decision making in a California registered and CLIA certified pathology lab, accredited by the Centers for Medicare & Medicaid Services. This study provides the first detailed characterization of the ex vivo heterogeneity of tumor responses in ovarian cancer samples exposed to NCCN‐recommended treatments using a functional profiling platform.

## 2 Materials and Methods

The ovarian cancer samples analyzed in this study were received both from R&D collaborations (de‐identified) and clinical samples (with written consent that specimen may be used for R&D purposes). All specimens were de‐identified prior to receipt by the investigators, and no identifiable private information was accessible to the investigators. Under 45 CFR 46.102(e) (1), human subjects research involves obtaining identifiable private information or biospecimens through interaction or intervention with a living individual. Because this study involved only analysis of de‐identified biospecimens and no interaction with patients, it did not meet the regulatory definition of human subject research. Therefore, Institutional Review Board (IRB) review and approval were not required. Surgically resected biopsies from 17 ovarian cancer patients were shipped overnight to SageMedic Corporation, (Redwood City, California), in a proprietary solution for shipment and storage using a temperature‐controlled transport kit that keeps the sample specimens at a temperature of 2 to 8°C to preserve the integrity of the tissue.

**FIGURE 1:**
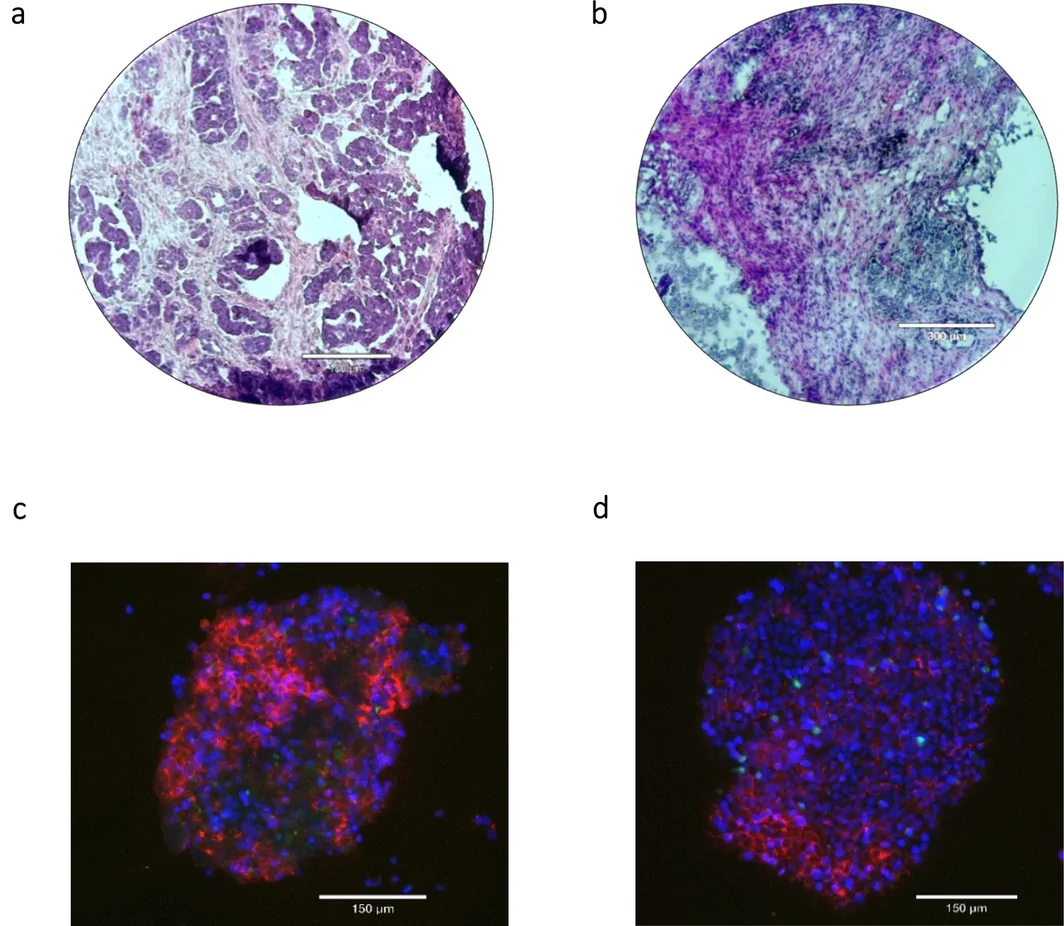
(a) H&E image from ovarian sample with a high‐grade serous carcinoma shows a moderately differentiated ovarian adenocarcinoma, consistent with endometrioid type, 20× magnification. (b) H&E image of an ovarian cancer tissue shows a poorly differentiated malignancy with sarcomatoid features, 10× magnification. (c) Immunofluorescence stains of 3D microtumors from a primary and (d) a metastatic ovarian cancer sample. Tissue 3D fragments were stained using Hoechst 33342 for the nuclei (blue), EpCAM as a tumor marker (red), and CD45 as a leukocyte marker (green). Notably most of these microtumors tended to be “cold”, i.e., having only a few immune‐competent cells.

A small portion of each of the tissue samples was frozen in optimal cutting temperature (OCT) compound (Cat #4583; Sakura Finetek, California, USA) for histological and morphological assessment. These sections were used to confirm tumor presence and tissue architecture and were not used for drug testing. 8 μm sections were generated using a cryotome (Shandon, Thermo Fisher Scientific, Massachusetts, USA), then mounted onto glass slides and dried at 37°C for 30 minutes before staining. After hydrating in distilled water for 2 minutes, cryosections were stained using the hematoxylin and eosin (H&E) protocol from Vectors Laboratories (Cat #H‐3502; Newark, California, USA). Images as shown in Figure 1a,b were taken using the Echo Revolve Inverted Microscope (Echo, California, USA).

Immunofluorescent stains (Figure 1c,d) were performed to detect the presence of epithelial (EpCAM) and leukocyte markers (CD45). The 3D microtumors were fixed using 4% paraformaldehyde (Cat #J19943.K2, ThermoFisher Scientific, Massachusetts, USA) for 10 minutes at room temperature followed by three washes with ice‐cold PBS. Samples were blocked for 30 min using a blocking buffer (3% Human Serum in PBS) at room temperature. Antibody solution containing 1:300 dilutions of AF647 Anti‐EpCAM (Cat# ab237385, RRID: AB_2940927) and AF488 Anti‐CD45 (Cat# ab40763; RRID: AB_726545) antibodies (Abcam, Cambridge, UK) was prepared using the blocking buffer. After 2 hours, samples were washed 3 times with ice‐cold PBS. Samples were counterstained with Hoechst 33342 (Cat #40044; Biotium, California, USA) and imaged using the Echo Revolve Inverted Microscope (Echo, California, USA).

Ovarian cancer specimens were processed on the day of receipt to maximize the yield of tumor cells and tumor fragments as described elsewhere [15] and cultivated to preserve the patient's cancer heterogeneity and 3D microenvironment as illustrated in the immunofluorescent stains. Following overnight incubation of the processed tissues at 37°C in a humidified incubator with 5% CO_2_, the 3D microtumors were dosed and incubated for 72 hours with a panel of up to 24 NCCN guideline‐recommended drugs and drug combinations, at standardized concentrations spanning at least three orders of magnitude, with each drug concentration tested in 4–5 replicates. Viable and non‐viable cells of the 3D cultures were detected using internally cross‐validated methods with the patient's own untreated cultures serving as the control. Image‐based methods such as the efficient quantification of viable and non‐viable cells were conducted using the ImageXpress Confocal Microscope (Molecular Devices, California, USA) as previously described [16]. Four‐parameter logistic dose–response curves were fitted using GraphPad Prism 10.4.1 and heterogeneity of the standardized EC_50_ drug concentrations was evaluated by the interdecile ratios. The interdecile ratio (IDR) was calculated as the ratio between the 90th and 10th percentiles of EC_50_ values for each drug and drug combination, providing a measure of variability while minimizing the influence of potential outliers. Drug efficacy was categorized based on the percentage of tumor cell viability reduction observed in the assay. Responses were classified using predefined thresholds as follows: efficacy > 75% was considered sensitive, efficacy between 25% and 75% was considered intermediate, and efficacy < 25% was considered resistant.

## 3 Results

The heterogeneity in tumor response was evaluated by processing and testing primary ovarian cancer tissues against a panel of therapeutic agents using an ex vivo functional profiling platform. The study included 17 ovarian cancer samples, with a mean patient age of 60.4 ± 16.7 years. The cohort included 76% White, 18% Black, and 6% South Asian patients. Histologically, the majority of tumors were high‐grade serous carcinomas (53%, *n* = 9), followed by low‐grade serous carcinomas (29%, *n* = 5), endometrioid carcinomas (12%, *n* = 2), and one granulosa cell tumor (6%). Most specimens represented advanced‐stage disease, frequently with peritoneal or omental metastases (47%). Most samples were obtained from primary tumors (88%, *n* = 15), while two samples were derived from metastatic lesions (12%, *n* = 2). The demographics and clinical characteristics of the patients included in this study are summarized in Table 1.

**TABLE 1:** Demographics and clinical characteristics.

Demographics	Mean/Type	Std/Occurrence level, %, (*n*)
Age (Years)	60.4	16.7
Gender	Female	100% (17)
Race	White	76% (13)
	Black	18% (3)
	South Asian	6% (1)

Clinical characteristics	Mean/Type	Std/Occurrence level, %, (*n*)
Tumor size (cm)	13	6.3
Histology Type	Serous Carcinoma, high grade	53% (9)
	Serous Carcinoma, low grade	29% (5)
	Endometrioid Carcinoma	12% (2)
	Granulosa cell tumor	6% (1)
pT Category	pT1	18% (3)
	pT3	53% (9)
	Unknown	29% (5)
pN Category	pN0	29% (5)
	pN1	12% (2)
	pNx	59% (10)
Other tissue involvement (multiple selections possible)	Peritoneal/Omental	47% (8)
	Genitourinal	41% (7)
	Intestinal	24% (2)
FIGO stage	IA	18% (3)
	IIIB	18% (3)
	IIIC	29% (5)
	Unknown	35% (6)
p53	Normal	24% (4)
	Abnormal (mutant pattern)	30% (7)
	Unknown	35% (6)
Biomarkers (multiple selections possible)	Epithelial, cytokeratins	18% (3)
	Epithelial, CK7	18% (3)
	Serous markers, WT‐1	35% (6)
	Serous markers, PAX8	29% (5)
	Cell cycle, p16	24% (4)
	Estrogen receptor, ER+	35% (6)

Histological examinations of ovarian cancers were performed using hematoxylin and eosin (H&E) staining to verify significant tumor presence (Figure 1b). Immunofluorescence analyses conducted on a patient's primary and metastatic tumor sample invariably revealed a substantial population of EpCAM‐expressing tumor cells, accompanied by a much smaller population of CD45‐positive leukocytes (Figure 1c,d). The detection of EpCAM^+^ tumor cells confirmed the epithelial origin and malignant nature of the samples, while the presence of CD45^+^ leukocytes reflected the presence of immune cells within the tumor microenvironment, underscoring the tumoral heterogeneity and complexity preserved within 3D microtumors.

To assess tumor response, the interdecile ratios (IDRs) of individual drugs and drug combinations were calculated to quantify variability in drug efficacy across the samples (Table 2).

**TABLE 2:** Standardized drug concentration metrics (lower concentrations reflect higher cytotoxicity as an outcome).

	Carboplatin	Paclitaxel	Gemcitabine	Doxorubicin	Olaparib	Carboplatin + Paclitaxel
Minimum	0.45	0.48	0.05	0.04	3.2	0.01
10th Percentile	0.45	2.4	0.07	0.06	4.0	0.01
25th Percentile	0.73	5.64	0.15	0.12	6.8	0.32
Median	1.1	8.3	0.24	0.44	18	0.43
Mean, geometric	1.5	10	0.85	0.32	15	0.81
75th Percentile	2.6	29	5.9	0.69	22	7.2
90th Percentile	24	100	100	1.3	65	56
Maximum	100	100	100	1.9	100	62
90th/10th Ratio	**54**	**42**	**1504**	**20**	**16**	**3880**

*Note:* Noteworthy, the ratios of the 90th to 10th percentiles differ widely from drug to drug.

**FIGURE 2:**
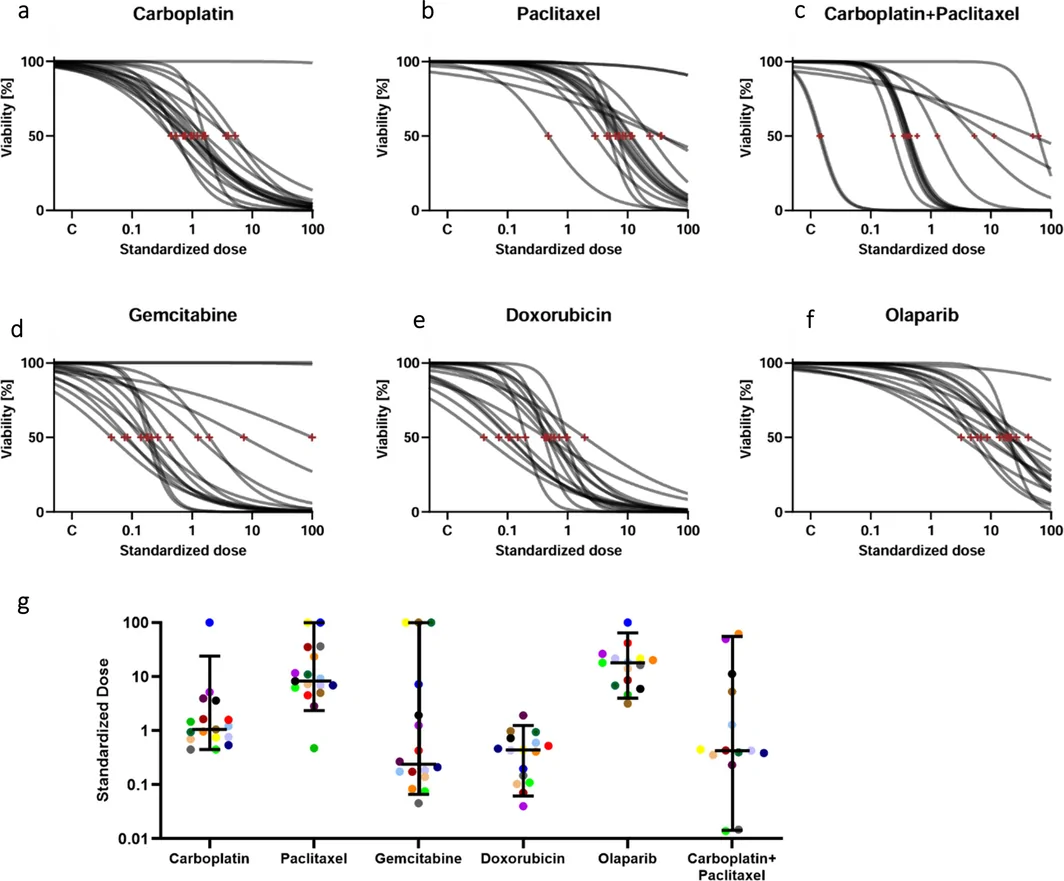
(a–f) Dose–response curves for cell viability following treatment with NCCN‐recommended drugs and drug combinations. Each curve represents one of the 17 individual samples included in this study, with cell viability measured as a percentage relative to untreated controls across a range of standardized doses. The red crosses indicate the EC_50_ values for each sample. (g) Scatter plot represents the distribution of the EC_50_ values for the 17 ovarian cancers showing the median and the 90th to 10th interdecile range. Each dot represents the EC_50_ value of an individual sample for some of the NCCN‐recommended therapies and combinations for ovarian cancer.

For the individual agents, carboplatin and paclitaxel (Figure 2a,b) exhibited IDRs of 54‐fold and 42‐fold, respectively, indicating moderate variability in tumor response. The combination of carboplatin and paclitaxel (Figure 2c) showed a significantly higher IDR of 3880‐fold, suggesting potential synergy between these agents in certain tissues but not in others. Gemcitabine (Figure 2d) demonstrated an IDR of 1504‐fold, reflecting high variability in its efficacy across the samples. In contrast, the responses to doxorubicin and olaparib (Figure 2d,f) were more consistent across samples, with relatively low IDRs of 20‐fold and 16‐fold, respectively. A two‐dimensional multivariate analysis of variance (MANOVA) on log‐transformed EC_50_ and Hill‐slope values (*n* = 97) confirmed a global drug effect (Pillai's trace = 0.69, *p* < 0.001), ruling out a shared multivariate distribution (Figure 2a–f). Focusing on potency alone, log‐transformed EC_50_ values remained heteroscedastic (Levene‐median *p* = 0.0376), and Welch's ANOVA test was significant (*p* < 0.0001). Games–Howell contrasts (Benjamini–Hochberg adjusted) identified 8 of 15 drug pairs with distinct EC_50_ distributions (Figure 2g). These findings underscore the substantial variability in therapeutic efficacy among patient‐derived ovarian cancer tissues, highlighting the importance of individualized therapy selection based on functional profiling rather than relying solely on standard treatment regimens.

**FIGURE 3:**
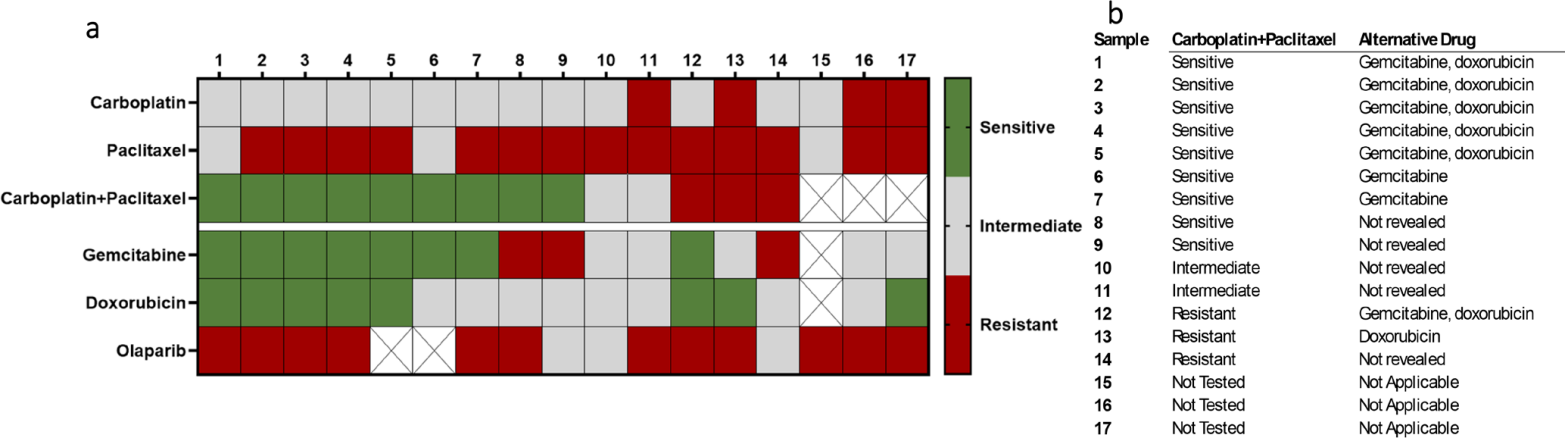
(a) Heatmap illustrating drug sensitivity across the ovarian cancer cohort based on efficacy values obtained from ex vivo drug testing. Each column represents an individual tumor sample, and each row represents a tested therapeutic agent or drug combination. Using predefined thresholds, responses were classified as sensitive (green) when efficacy exceeded 75%, intermediate (gray) when efficacy ranged from 25% to 75%, and resistant (red) when efficacy was below 25%. Samples that were not tested for a specific drug are represented with a cross. (b) Relative sensitivity classification for the carboplatin–paclitaxel combination and alternative agents demonstrating higher sensitivity in tested samples.

To further explore potential clinical implications of the observed heterogeneity, samples tested with the standard of care carboplatin–paclitaxel combination were categorized based on their treatment efficacy. Among the 14 evaluable specimens, 64% (*n* = 9) were classified as sensitive, 14% (*n* = 2) demonstrated intermediate sensitivity, and 21% (*n* = 3) were classified as resistant according to the predefined efficacy thresholds (Figure 3a). Of note, several samples demonstrated higher sensitivity to the carboplatin–paclitaxel combination rather than to the single agents. In specimens classified as resistant or intermediate to the carboplatin–paclitaxel combination, other agents demonstrated comparatively higher sensitivity (Figure 3b).

## 4 Discussion

This study demonstrated significant interpatient heterogeneity of primary ovarian cancer tissues using a novel ex vivo functional profiling platform. Among the 17 ovarian cancer samples analyzed, interpatient heterogeneity was evident across numerous NCCN‐recommended therapies. For individual agents, carboplatin and paclitaxel exhibited moderate variability in response, with interdecile ratios (IDRs) of 52‐fold and 42‐fold respectively. However, their combination displayed substantially higher heterogeneity (IDR = 3880‐fold), suggesting synergy in some samples but not in others. Additionally, several tumors were more sensitive to the carboplatin–paclitaxel combination than to either single agent alone, underscoring the importance of assessing both individual drugs and clinically relevant combinations. Gemcitabine alone showed the highest variability (IDR = 1504‐fold), reflecting a wide range of patient‐specific responses, whereas doxorubicin and olaparib demonstrated relatively consistent responses (IDRs = 20‐fold and 16‐fold, respectively), albeit with limited efficacy in most samples.

Statistical analysis of EC_50_ distributions confirmed substantial heterogeneity, as indicated by Welch's ANOVA (*p* < 0.0001) and Games–Howell post hoc tests, which identified significant differences in 8 of 15 drug pairs (Benjamini–Hochberg adjusted *p* < 0.05). These findings align with the clinically observed variability in patient treatment responses, emphasizing the importance of individualized therapy selection. Importantly, this platform's ability to quantify tumor heterogeneity offers a valuable tool for personalized oncology. By capturing variations in tumoral responses, it provides actionable insights that may guide oncologists in tailoring therapies based on an individual patient's tumor profile rather than relying solely on genetic markers. This approach could be particularly beneficial for patients with tumors exhibiting high heterogeneity or resistance to standard treatments, allowing for more precise therapeutic selection. Notably, several samples classified as resistant to platinum–taxane therapy exhibited sensitivity to gemcitabine and doxorubicin, agents frequently used as second‐line treatments in recurrent or platinum‐resistant ovarian cancers [17].

### 4.1 Tumor Microenvironment

This study highlights a critical gap in current precision oncology approaches: the limitations of relying solely on static molecular data to guide treatment decisions. While sequencing technologies have revealed tumor heterogeneity and evolution, as well as actionable mutations in some cases, the majority of patients cannot be matched to effective therapies [18].

A key reason for this gap lies in the tumor microenvironment (TME), which plays a crucial role in influencing tumor resistance and therapeutic outcomes [19]. The TME encompasses a dynamic network of stromal cells, immune components, extracellular matrix, hypoxia, and nutrient gradients, all of which interact with cancer cells to modulate drug sensitivity and resistance [20]. These factors are not adequately represented by genomic, transcriptomic, proteomic, metabolic, or other biomarker‐based analyses, which often provide only partial snapshots of tumor biology [12].

Viable tissue‐based assays, such as the functional profiling platform described here, address this limitation by preserving tumor architecture and the influence of the TME. By maintaining cell–cell and cell‐matrix interactions in 3D microtumors, including pan‐leukocytes, as shown in Figure 1c,d, this platform captures a tumor's live response to therapeutic agents, offering actionable insights that molecular diagnostics alone cannot provide. These findings emphasize the need for integrated approaches that combine molecular and functional profiling to better account for the multifactorial nature of drug response, ultimately bridging the gap between biomarker discovery and improved patient outcomes.

### 4.2 Clinical Relevance

These findings highlight the critical role of functional profiling in addressing the clinical challenges of advanced ovarian cancer, particularly in personalizing therapeutic decisions, such as identifying carboplatin‐paclitaxel‐resistant tumors in the neo‐adjuvant setting. While neo‐adjuvant therapy is increasingly used to optimize surgical outcomes, patients with resistant tumors face delayed surgery and unnecessary toxicities from ineffective chemotherapy [3]. Functional profiling could preemptively identify those tumors, enabling oncologists to pursue immediate surgery or alternative therapeutic strategies without significant delays [3]. These alternative strategies could include agents such as gemcitabine, doxorubicin, or PARP inhibitors, the latter specifically if genomic data are available. By quantifying tumor‐specific responses, functional profiling provides actionable insights to improve outcomes in a patient‐specific manner, reducing the reliance on trial‐and‐error approaches [19, 21]. By delivering real‐time, patient‐specific data on tumor sensitivities within a 10‐day time frame, this platform not only optimizes therapy selection within standard of care but also facilitates enrollment in clinical trials for experimental or off‐label treatments. In fact, short‐turnaround functional drug sensitivity testing on patient‐derived tumor material has been increasingly explored to complement genomic profiling, with multiple studies demonstrating clinically actionable reporting timelines and integration into treatment decision‐making workflows [13, 22]. Because tumor specimens can be shipped to specialized laboratories using temperature‐controlled transport kits, this approach may enable participation from a wide range of clinical facilities, including centers without on‐site functional testing capabilities. Its integration into clinical workflows could transform ovarian cancer management, improve survival rates and quality of life while minimizing unnecessary toxicities from ineffective treatments.

### 4.3 Opportunities for Functional Profiling

The relatively small sample size of 17 ovarian cancer tissues limits the generalizability of these findings; however, finding such a marked heterogeneity of tumor responses even within this limited cohort supports furher evaluation of this approach in larger studies.

A major strength of this platform is its ability to perform comprehensive drug testing with minimal tissue requirements. The typical workflow for late‐stage patients requires an additional biopsy, whereas we can evaluate all key drugs using just 2–3 samples from a 16 G core needle biopsy. In many cases, tumor tissue can be retrieved from ascites, providing a less invasive and viable alternative sampling method.

This platform generates detailed analytics within a 10‐day time frame, providing oncologists with timely information about the sensitivity and resistance profiles of the patient's tumor to standard NCCN‐guideline therapeutics. Moreover, quality control studies have demonstrated that the assay results remain highly reproducible, even with variability in sample collection and shipment times of 2–3 days.

While ex vivo drug responses of this platform have not yet been correlated with clinical outcomes such as tumor response or progression‐free survival due to de‐identified research samples and the unavailability of 3–12 months follow‐ups for many clinical samples, two key factors support the platform's relevance. First, cancers that can withstand 10× pharmacological doses ex vivo are highly unlikely to respond clinically. Second, the platform provides estimates of relative drug sensitivity by comparing tumors to other specimens in the cohort, allowing a classification of the tumor samples as more or less sensitive compared to others.

### 4.4 Future Directions

Future efforts will focus on validating the platform by correlating ex vivo findings with clinical outcomes and integrating it into prospective clinical trials. Including molecular profiling data and comparing those with the functional profiling results will provide valuable complementary insights. Furthermore, it is also possible to include drugs that are in development so that the platform might serve as a guidance for cancer patients who have exhausted all options, steering them toward clinical trials with drugs most likely to yield a clinical benefit.

## 5 Conclusions

This study demonstrates the potential of an ex vivo functional profiling platform to address the challenges of therapeutic heterogeneity in advanced ovarian cancer. By preserving the tumor microenvironment and quantifying drug responses across a wide range of concentrations within a ten‐day time frame, this platform provides actionable insights that can guide personalized therapy selection. Importantly, it enables oncologists to optimize treatment strategies using NCCN‐recommended therapies, reducing exposure to ineffective treatments and improving clinical decision‐making.

The ability to identify carboplatin‐paclitaxel resistance prior to neo‐adjuvant therapy, select appropriate alternatives following platinum failure, and evaluate options for multidrug‐resistant tumors underscores the platform's utility to provide superior care within the existing standard of care framework. As this approach continues to be refined and validated, it has the potential to transform the management of ovarian cancer, improving patient outcomes while maintaining alignment with current clinical guidelines.

## Author Contributions

C.M. acquired data, analyzed data, interpreted data, prepared figures (1, 2) and tables (1, 2), and wrote the main manuscript text. I.T. analyzed and interpreted data, prepared Table 2 and Figure 2, and wrote the manuscript text. R.N. and J.K. acquired data, prepared Table 1, and wrote the manuscript text. R.J.P. supervised the research, wrote and revised the manuscript text. O.D.T., and W.C. wrote the manuscript text and revised it. C.A. conceived the study, supervised the research, interpreted the data, wrote the manuscript text, and provided funding. All authors reviewed and approved the final manuscript.

## Funding

This work was supported by the SageMedic Corp.

## Conflicts of Interest

The technology reported in this study was developed by SageMedic Corp. Chiara Maestri, Rajeshwar Nitiyanandan, and Christian Apfel are SageMedic employees. Ivan Trus, Ricardo J. Parker, and William Cance are independent contractors at SageMedic. William Cance and Christian Apfel serve as fiduciary officers. Christian Apfel reports ownership of patents related to the presented technology.

## Data Availability

The data generated and analyzed in this study derived from de‐identified ovarian cancer specimens obtained from research and clinical collaborators. Data is not publicly available but may be shared upon request from the corresponding author and is subject to appropriate approvals and data sharing agreements.
